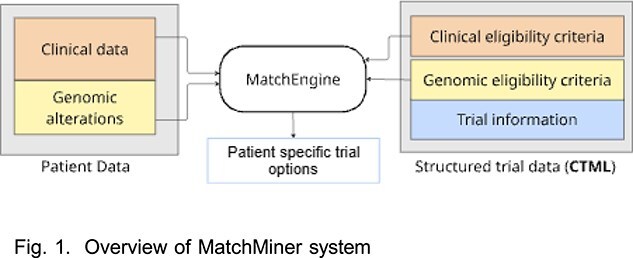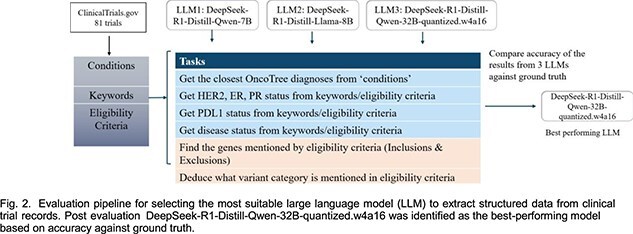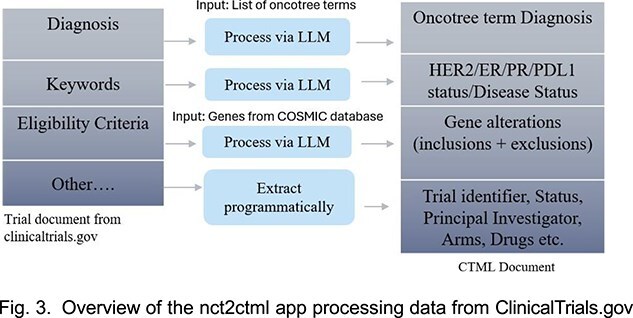# AI-assisted patient matching for personalized cancer medicine

**DOI:** 10.1093/bib/bbaf631.013

**Published:** 2025-12-12

**Authors:** Sumedha Saxena, Edmond S K Ma, Aya El Helali, David J H Shih

**Affiliations:** School of Biomedical Science, Li Ka Shing Faculty of Medicine, The University of Hong Kong, Hong Kong SAR, China; Division of Clinical Pathology & Molecular Pathology, Hong Kong Sanatorium & Hospital, Hong Kong SAR, China; Department of Clinical Oncology, Centre of Cancer Medicine, School of Clinical Medicine, Li Ka Shing Faculty of Medicine, The University of Hong Kong, Hong Kong SAR, China; Center of Oncology and Immunology, University of Hong Kong; School of Biomedical Science, Li Ka Shing Faculty of Medicine, The University of Hong Kong, Hong Kong SAR, China; Center of Oncology and Immunology, University of Hong Kong

## Abstract

**Background:**

Advanced sequencing techniques have facili- tated the implementation of cancer precision medicine, in which treatments are targeted against specific genomic alter- ations. To advance the development of precision oncology drugs, it is important to improve the participation of can- cer patients in clinical trials. In this context, the Molecular Tumor Board (MTB) plays a crucial role in facilitating the matching of patients with appropriate clinical trials [1], but some challenges remain unresolved. Trials can have complex eligibility criteria that are often presented in an unstructured format, making it difficult to extract key information for checking patient eligibility. Similarly, patient data are often in an unstructured format such as free text, tabular data, and images. Disease diagnoses in clinical notes also do not use standardized terminologies. These technical challenges complicate patient-trial matching.

The primary objective of this project is to match cancer patients in Hong Kong to the clinical trials in Hong Kong and nearby regions, with better accuracy and efficiency. To achieve this, we seek to harmonize patient data from multiple sources and formats and convert them to a structured format. Additionally, we aim to harmonize clinical trials and their eligibility criteria from different regions, facilitating a more efficient patient-trial matching process, which aligns with the recent regional consensus on precision oncology practices [2].

Methodology:

**Deployment of the MatchMiner application:**

We set up MatchMiner, an open-source platform developed by Dana- Farber Cancer Institute, to match patient-specific genomic and clinical data with trial eligibility criteria [3]. It employs MatchEngine to match patient-trial data and produce patient specific trial options. Fig. 1 shows the overview of the Match- Miner system.

**Customization of MatchMiner for the local context:**

We extended MatchMiner’s matching platform to include additional clinical criteria, including HER2, ER, PR, and PD- L1 status. To standardize diagnosis, we use the stable release 2025___04___08 of the OncoTree ontology [4]. We further refined the variant classification for genomic criteria matching.

**Data extraction with LLMs:**

To convert patient data and trial eligibility criteria into a structured format, we evaluated multiple large language models (LLMs) for data extraction tasks. Key tasks include mapping diagnoses to OncoTree terms and extracting biomarkers and genomic criteria from the unstructured text in trial eligibility criteria. As input, we pulled 81 trials from ClinicalTrials.gov and prepared ground truths for the tasks mentioned above. We engineered prompts for all tasks and tested them with multiple LLMs, comparing the results based on timing and correctness against ground truth. Based on results, **DeepSeek-R1-Distill-Qwen- 32B-quantized.w4a16** was selected for its superior accuracy, and used subsequently for extracting information from raw data. Fig. 2 summarizes the tested models and associated tasks.

**Web interface for patient data entry:**

To capture patient data, we developed a web-based user interface application (**matchminer-patient**) that facilitates the input and conversion of the following data:

- **Clinical data:** Diagnosis and patient history in free-form text, which will be processed by LLMs to identify an OncoTree diagnostic term and extract disease character- istics.
- **Genomic data:** Clinical sequencing results in the form of screenshot images. We use SuryaOCR to extract text from each image, which is then processed by LLMs to identify actionable genes and genetic alterations. Images are deleted immediately to ensure that no protected health information (PHI) is stored. The output is structured patient data compatible with MatchMiner.

**Harmonization of clinical trial data:**

To harmonize clinical trial data, we developed **nct2ctml**, an application that retrieves data from ClinicalTrials.gov and other international registries, facilitating local synchronization of eligibility cri- teria and status in our database. Fig. 3 shows the workflow for processing trial documents from ClinicalTrials.gov and converting to CTML format. The process involves:

1) **Data extraction:** Extract key trial details such as free- text diagnoses, eligibility criteria in terms of clinical and genomic features.
2) **LLM-based processing:** Use a locally hosted LLM to convert unstructured trial data into structured formats by mapping free-text diagnoses to OncoTree terms, extracting biomarkers like HER2, ER, PR, and PD-L1 with their values from eligibility criteria, and identifying inclusion and exclusion criteria for gene alterations using a curated COSMIC gene list.

The output of **nct2ctml** is a **Clinical Trial Markup Language**

(CTML) document containing metadata such as trial status, principal investigator, trial arms, drugs along with structured clinical and genomic criteria of the trial.

**Synchronization of local and international trial reg- istries:**

To keep our local database synchronized, we continu- ously aggregate trial data from local sources like HKU Clinical Oncology, Medical Oncology, HKU Clinical Trial Registry, and CUHK’s Comprehensive Cancer Trial Unit—as well as from international repositories like ClinicalTrials.gov (US), the EU Clinical Trials Register, and national registries in South Korea, Singapore, and Taiwan. A key challenge is that local trial registries usually do not include international identifiers, which necessitates manual lookup and curation to match local trials to international trial registries. Our **nct2ctml** application also detects duplicates and merges local and international trial data in the database.

**Results:**

We harmonized data for 124 cancer clinical trials in Hong Kong and nearby regions, converting them into CTML format for MatchMiner. AI-assisted CTML creation required manual intervention in approximately 11% of trials.

Patient data from multiple sources — including sequencing reports from multiple vendors and free-form clinical notes — were integrated and converted to PHI-free structured data. AI- assisted generation of structured patient data required manual intervention in about 30% of cases.

With our aggregated clinical trial database, MatchMiner generates 7–10 trial matches on average per patient. Beginning in Q1 2025, these trial matches are being reviewed at monthly MTB meetings to support personalized treatment planning.

**Conclusion:**

We developed a suite of AI-enabled software tools to collect unstructured patient and clinical trial data and convert them into structured formats, enabling effective matching of cancer patients to relevant clinical trials. This software suite supports multidisciplinary MTBs by providing actionable insights for personalized treatment planning and clinical trial enrollment.

**Code availability:**

Source code for this project is available at-

- https://github.com/sumedhasaxena/matchminer-setup
- https://github.com/sumedhasaxena/nct2ctml
- https://github.com/sumedhasaxena/matchminer-patient

**Acknowledgment:**

This work was supported by the Health and Medical Research Fund, the Health Bureau, The Govern- ment of the Hong Kong Special Administrative Region (project 11,222,156).

**References:**

1. El Helali A, Lam TC, Ko EY, Shih DJH, et al. ‘The impact of the multi- disciplinary molecular tumour board and integrative next generation sequencing on clinical outcomes in advanced solid tumours.’ Lancet Reg Health West Pac. 2023;36:100775.

2. Lam TC, Cho WC, Au JS, Ma ES, Lam ST, et al.; Precision Oncology Working Group (POWG). ‘Consensus statements on precision oncology in the China Greater Bay Area.’ JCO Precis Oncol. 2023;7:e2200649.

3. Klein H, Mazor T, Siegel E, et al. ‘MatchMiner: an open-source platform for cancer precision medicine.’ npj Precis Onc. 2022;6:69.

4. Smyth LM, et al. ‘OncoTree: a cancer classification system for precision oncology.’ JCO Clin Cancer Inform. 2021;5:221–230.